# Bone-forming capacity of adult human nasal chondrocytes

**DOI:** 10.1111/jcmm.12526

**Published:** 2015-02-16

**Authors:** Benjamin E Pippenger, Manuela Ventura, Karoliina Pelttari, Sandra Feliciano, Claude Jaquiery, Arnaud Scherberich, X Frank Walboomers, Andrea Barbero, Ivan Martin

**Affiliations:** a Departments of Surgery and of Biomedicine, University Hospital Basel, University of BaselBasel, Switzerland; b Department of Biomaterials, Radboud University Nijmegen Medical CentreNijmegen, The Netherlands

**Keywords:** nasal chondrocytes, craniofacial bone, intramembranous ossification, preclinical studies, stromal cells

## Abstract

Nasal chondrocytes (NC) derive from the same multipotent embryological segment that gives rise to the majority of the maxillofacial bone and have been reported to differentiate into osteoblast-like cells *in vitro*. In this study, we assessed the capacity of adult human NC, appropriately primed towards hypertrophic or osteoblastic differentiation, to form bone tissue *in vivo*. Hypertrophic induction of NC-based micromass pellets formed mineralized cartilaginous tissues rich in type X collagen, but upon implantation into subcutaneous pockets of nude mice remained avascular and reverted to stable hyaline-cartilage. In the same ectopic environment, NC embedded into ceramic scaffolds and primed with osteogenic medium only sporadically formed intramembranous bone tissue. A clonal study could not demonstrate that the low bone formation efficiency was related to a possibly small proportion of cells competent to become fully functional osteoblasts. We next tested whether the cues present in an orthotopic environment could induce a more efficient direct osteoblastic transformation of NC. Using a nude rat calvarial defect model, we demonstrated that (*i*) NC directly participated in frank bone formation and (*ii*) the efficiency of survival and bone formation by NC was significantly higher than that of reference osteogenic cells, namely bone marrow-derived mesenchymal stromal cells. This study provides a proof-of-principle that NC have the plasticity to convert into bone cells and thereby represent an easily available cell source to be further investigated for craniofacial bone regeneration.

## Introduction

Cells from the adult nasal septum (nasal chondrocytes, NC) derive from the same multipotent embryological segment that gives rise to the majority of the bone and cartilage of the head and face (neural crest/neuroectoderm) 1. Human septal cartilage has long been considered the pacemaker for the growth of the face and skull, with growth potential equivalent to that of the epiphyseal growth cartilage of long bones 2. Adult nasal septal tissue is known to directly interface with facial bone tissue development, thereby regulating turbinate and maxilla bone growth 3. *In vitro* studies have demonstrated that NC retain a certain level of plasticity and can acquire traits of neuronal- and osteoblast-like phenotypes 4,5. However, it is unknown if NC can induce or directly from frank bone tissue *in vivo*.

There are two archetypal routes for bone formation and repair: endochondral and intramembranous ossification 6. The primary route in bone development and repair is endochondral ossification, in which a hypertrophic cartilage template is formed and eventually remodelled into bone tissue. The bones of the head and face form and repair predominantly through intramembranous ossification. Since NC reside within cartilage tissue and are in direct contact with an environment that forms through intramembranous ossification, in principle they could have the capacity to form bone through either ossification route.

In this study, we assessed the capacity of adult human nasal septum-derived chondrocytes, appropriately primed *in vitro* through hypertrophic or osteoblastic differentiation, to form bone tissue *in vivo*. NC were cultured also at clonal levels under osteoblastic differentiation conditions to determine the extent of heterogeneity of their *in vitro* osteogenic potential. Exploiting both subcutaneous and orthotopic cranial *in vivo* environments, we then assessed whether human NC could be phenotypically converted to osteoblasts and actively participate in the formation of frank bone tissue. The relative easy availability of a craniofacial-derived somatic cell source 7, capable of active participation in homotopic bone repair without pre-implantation genetic manipulation, would provide a significant and clinically relevant advancement in the field of craniofacial bone repair.

## Materials and methods

### Methods

#### Cell source and expansion

All cell sources were obtained in accordance with the local ethical committee (University Hospital Basel) and subsequent to informed consent. Nasal septal biopsies were harvested using a punch biopsy tool (6 mm diameter) by first inserting and gently pushing it into the cartilage, taking care not to perforate the opposite side. A total of 3 biopsies from 3 different donors (aged from 52 to 76 years old; two females and one male) were obtained in this manner and used for subsequent experiments. Articular cartilage tissues were harvested post-mortem from full-thickness biopsies of the femoral condyle of 3 patients (aged from 43 to 67 years old; two males and one female). Chondrocytes (both NC and articular chondrocytes, AC) were isolated by enzymatic digestion using collagenase II (Worthington, USA) according to an established protocol 8 and expanded for two passages (corresponding to approximately 7 population doublings) in DMEM complete medium [CM; containing 10% foetal bovine serum, 100 mM HEPES buffer solution, 1 mM sodium pyruvate, 100 U/ml penicillin, 100 mg/ml streptomycin and 292 mg/ml l-glutamine (Gibco, Basel, Switzerland)] supplemented with 5 ng/ml Fibroblast Growth Factor-2 (FGF-2; R&D Systems, Minneapolis, USA). Medium was changed twice a week. Upon confluence, cells were enzymatically retrieved, counted and used as described below. Human bone marrow-derived mesenchymal stromal cells (BMSC; used as a control throughout this study) were isolated from marrow aspirates (volume ∽20 ml) obtained from the iliac crest 9 of three patients (37–45 years old, 2 males and 1 female) and expanded for two passages (corresponding to approximately 14 cell population doublings) in α-Modified Eagle's Medium (αMEM) based CM in the presence of 5 ng/ml FGF-2 to enhance their post-expansion bone forming capacity 10.

#### Cell-based construct fabrication and culture

Constructs recapitulating the endochondral ossification route were established using a previously described pellet culture system 11. Briefly, cells (500,000) were centrifuged in 1.5 ml conical polypropylene tubes (Sarstedt, Numbrecht, Germany) to form spherical pellets. Pellets were then cultured in serum free medium (SFM) consisting of DMEM, 4.5 mg/ml d-Glucose, 0.1 mM nonessential amino acids, 1 mM sodium pyruvate, 100 mM HEPES buffer, 100 U/ml penicillin, 100 mg/ml streptomycin, 0.29 mg/ml l-glutamine and Insulin-Transferrin-Selenium (ITS+1) supplemented with 0.1 mM ascorbic acid 2-phosphate, 10 ng/ml transforming growth factor-β1 (TGF-β1) and 10^−8^ M dexamethasone 12. After 3 weeks of chondrogenic culture, medium was changed to hypertrophic induction medium, which consisted of SFM supplemented with 0.1 mM l-ascorbic acid 2-phosphate, 10^−8^ M dexamethasone, 50 nM thyroxine and 10 mM β-glycerophosphate 13. For each experiment and experimental group, at least 2–3 replicate pellets were assessed for each analysis.

Constructs recapitulating intramembranous ossification were established by loading 1 million expanded cells after one passage (average of 4.5 doublings) onto porous calcium phosphate granules (Actifuse™Microgranules, Apatech, Foxborough, MA, USA) using fibrin gel as a cell carrier, as previously described 14. To obtain the fibrin mesh, fibrinogen and thrombin components from Tisseel VH S/D (Baxter BioScience, Vienna, Austria) were diluted and mixed as described previously 15. The fibrinogen component (containing 75–115 mg/ml fibrinogen, +3000 KIE/ml aprotinin) and the thrombin component (containing 400–600 IU/l thrombin + 40 mmol/l calcium chloride) were diluted three and fourfold, respectively, using the specific buffers provided by Baxter. All constructs were maintained in osteogenic medium for 2 weeks before being either ectopically or orthotopically implanted. Osteogenic medium consisted of αMEM CM supplemented with 10^−8^ M dexamethasone, 0.1 mM l-ascorbic acid-2-phosphate and 10 mM β-glycerolphosphate. The above experimental procedure was applied for both NC and BMSC and for both cell types under equivalent conditions.

#### Clonal production

Nasal chondrocytes were isolated and clonal populations generated according to a previously established procedure 16. Briefly, cells were extracted from the native nasal septum tissue biopsy (whole population). Single cells from this whole population were plated into one well of a 96-well plate and expanded in DMEM CM supplemented with 1 ng/ml TGF-β1 and 5 ng/ml FGF-2 17. Colonies deriving from a single cell were expanded for 3 passages (corresponding to approximately 22 doublings) (clonal population) before testing their osteoblastic differentiation capacity in established *in vitro* assays. Individual colonies were also tested *in vivo* for 8 weeks in subcutaneous pouches using the intramembranous ossification constructs described above.

#### Animal models

All *in vivo* procedures were performed in accordance with the standards and protocols of both the University Hospital Basel, Switzerland and Radboud University Nijmegen Medical Centre, Nijmegen, the Netherlands. National guidelines for care and use of laboratory animals were followed and approval was obtained from the country's governing body in which the experimentation occurred.

For subcutaneous implantations, constructs were implanted into the subcutaneous tissue of nude mice (CD1 nu/nu, athymic, 6–8 week-old females, Charles River, Sulzfeld, Germany). Constructs from the same experimental group were implanted in different mice, with up to four constructs implanted per mouse. A total of 6 replicates per experimental group resulted in the use of 5 mice. For orthotopic implantations, bilateral calvarial defects were made in 4 week-old, male nude rats (Crl:NIH-Foxn1rnu, Charles River) as previously described 18. Constructs were placed into the defects and molded to increase as much as possible the bone to construct contact. Finally, the periosteum and the scalp were closed with 3.0 and 4.0 Vicryl® resorbable sutures (Johnson & Johnson, St. Stevens-Woluwe, Belgium). For both subcutaneous and orthotopic implantations, constructs remained *in vivo* for a total of 12 weeks, whereupon the mice or rats were killed by inhalation of CO_2_. The constructs were harvested, fixed in 1.5% paraformaldehyde overnight and processed as described below. Redundant. Already described in first paragraph.

### Analytical methods

#### Histology

Explanted constructs were fixed overnight in 1.5% paraformaldehyde at 4°C. Ceramic-based constructs were then subjected to slow decalcification in 7% w/v EDTA and 10% w/v sucrose (both from Sigma-Aldrich, Saint Louis, USA) at 37°C on an orbital shaker for 7–10 days. Decalcification solution was refreshed daily. All constructs were then paraffin embedded (TPC15 Medite, Burgdorf, Germany), sectioned (6-μm-thick) by means of a microtome (Leica, Wetzlar, Germany) and processed for histological, histochemical and immunohistochemical stainings as follows. Standard haematoxylin and eosin (Baker) staining was performed to identify bone tissue formation and maturation stage. According to conventional definitions and in contrast to mere condensation of collagenous structures, frank bone tissue was identified as uniform eosin-pink stained regions, often including a visible rim of lining osteoblasts. Based on haematoxylin and eosin stained sections, quantification of total *de novo* bone area was performed based on 3 different depth sections of 3 different explants (*n* = 9). Total bone area was calculated automatically by totaling the sum of all traced contours of *de novo* bone spots using CellSense Dimension software (Olympus, Tokyo, Japan). Safranin-O (Fluka, Buchs, Switzerland) staining allowed investigating the presence of sulphated proteoglycans inside the construct, characteristic of cartilaginous tissue. The matrix characterization in cartilaginous constructs was assessed by immunostaining for human collagen type II (COLL II) and collagen type X (COLL X) (MP Biomedicals LLC, Santa Ana, USA and Abcam PLC, Cambridge, UK respectively). Upon rehydratation in ethanol series, sections were treated as described previously for antigen retrieval for COLL X according to the manufacturer's instructions 13. The presence of vessels was detected by immunostaining for CD31 (Abcam 28364). The immunobinding was detected with biotinylated secondary antibodies and using the appropriate Vectastain ABC kits (Vector Laboratories, Burlingame, USA). The red signal was developed with the Fast Red kit (Dako Cytomation Dako, Glostrup, Denmark) and sections counterstained by Haematoxylin. Negative controls were performed during each analysis by omitting the primary antibodies. Human cells in the explants were identified by chromogenic *in situ* hybridisation for the human-specific sequence ALU, using a biotin-conjugated DNA probe (ZytoVision GMBH, Bremerhaven, Germany), as per the manufacturers guidelines. Histological and immunohistochemical sections were analysed using an Olympus BX-61 microscope.

#### Calcium and DNA quantification

Extracellular matrix associated calcium present on all applicable constructs was determined colorimetrically on a Spectra Max 190 microplate colorimeter (Molecular Devices, Sunnyvale, USA) as previously described 19 and as per the manufacturer's protocol (Total Calcium Assay, Randox, Crumlin, UK). Total calcium was normalized to total DNA present in a replicate construct.

To quantify total DNA per construct, collected samples were digested with proteinase K solution (1 mg/ml proteinase K, 50 mM TRIS, 1 mM EDTA, 1 mM iodoacetamide, and 10 mg/ml pepstatin-A (Sigma-Aldrich) in double distilled water or potassium phosphate buffer for 16 hrs at 56°C as previously described 20. DNA quantification was performed by means of CyQUANT® Cell Proliferation Assay (Invitrogen, Waltham, USA). Working solutions were prepared according to the manufacturer's protocols. The analyses were carried out measuring fluorescence with a Spectra Max Gemini XS Microplate Spectrofluorometer (Molecular Devices). Excitation and emission wavelengths were, respectively, 485 and 538 nm. Samples in each plate included a calibration curve. Each sample was measured in triplicate.

#### Real-time PCR

Total RNA extraction, cDNA synthesis and real-time reverse transcriptase-polymerase chain reaction (RT-PCR; 7300 AB Applied Biosystems, Foster City, USA) were performed to quantify expression levels of the following genes of interest: COLL II, COLL X, bone sialoprotein (BSP), osteocalcin (OC) 16, collagen type I (COLL I), osteopontin (OP), osteonectin (ON), RUNX2, bone morphogenetic protein 2 (BMP2), bone morphogenic protein 4 (BMP4-Applied Biosystems Ref. number: Hs00181626_m1), Matrix Metalloproteinase-13 (MMP13-Applied Biosystems, Ref. number: Hs00233992_m1), alkaline phosphatase (ALP-Applied Biosystems Ref. number: Hs01029144_m1), osterix (SP7-Applied Biosystems, Ref. number: Hs00541729_m1), indian hedgehog homolog (IHH-Applied Biosystems, Ref. number: Hs01081800_m1), glioma associated oncogene homolog-1 (GLI1-Applied Biosystems, Ref. number: Hs00171790_m1), parathyroid hormone 1 receptor (PTH1R-Applied Biosystems, Ref. number: Hs00174895_m1), bone morphogenetic protein 4 (BMP-4, Applied Biosystems, Ref. number: Hs00181626_m1), and chondromodulin (LECT1, Applied Biosystems, Ref. number: Hs00993254_m1). Glyceraldehyde 3-phosphate dehydrogenase (GAPDH) was used as housekeeping, reference gene.

#### Statistics

Data are presented as mean ± SD; N is indicated in the figure legends. For statistical testing one-way anova was performed for multi-condition experiments using the Graphpad Prism software (Version 5.02, Graphpad Software, San Diego, USA).

## Results

### *In vitro* hypertrophic NC form stable cartilage *in vivo*

**Figure 1:**
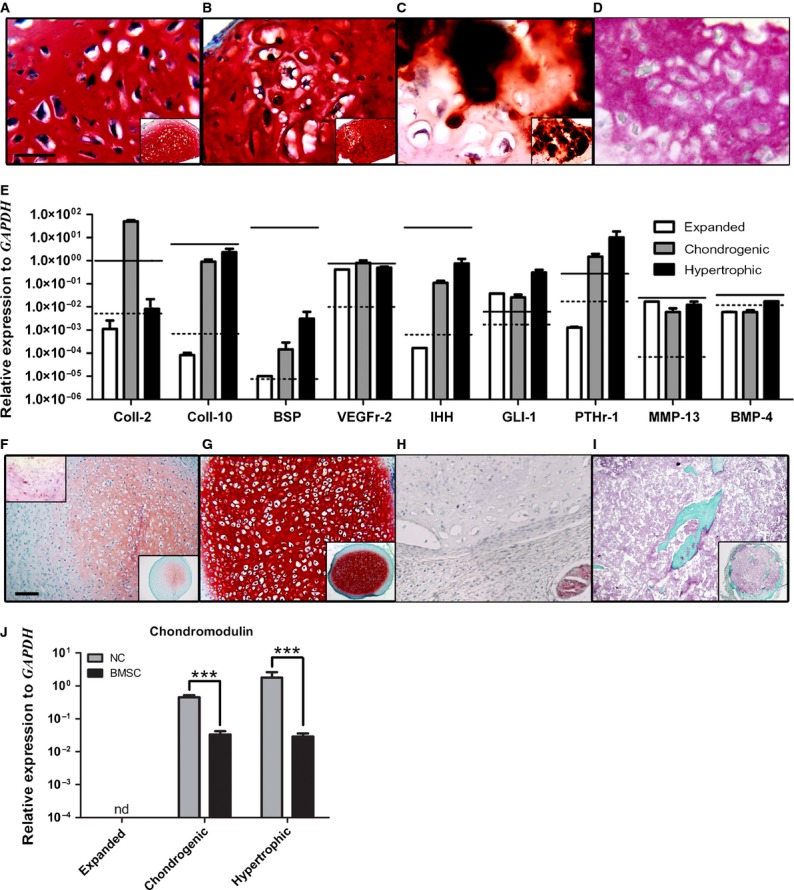
NC can form hypertrophic-like tissue *in vitro*, but revert to stable cartilage *in vivo*. *In vitro analysis*: Safranin-O staining of (A) chondrogenic- and (B) hypertrophic-induced NC constructs with (C) corresponding alizarin red staining of hypertrophic-induced NC constructs. (D) Immunohistochemistry staining of collagen type X in hypertrophic-induced NC constructs. (E) Gene expression analysis of NC (bars) compared to hypertrophic-induced AC (black dotted lines) and BMSC (black solid lines); *n* = 3. *In vivo analysis*: (F) Safranin-O and Collagen type X (upper left insert) staining of NC-based *in vitro* hypertrophic-induced constructs explanted after 5 weeks. (G) Safranin-O staining of NC-based *in vitro* hypertrophic-induced constructs explanted after 12 weeks subcutaneous implantation. (H) Immunohistochemistry staining of CD31 in 12 week explants. (I) Masson's trichrome staining of BMSC-based *in vitro* hypertrophic-induced constructs explanted after 12 weeks subcutaneous implantation. (J) Real-time RT-PCR analyses carried out using specific primers for chondromodulin expressed by expanded NC and NC cultured under chondrogenic or hyperthrophic conditions. Levels are normalized to glyceraldehyde 3-phosphate dehydrogenase (GAPDH). Values are mean ± SD of *n* = 3 donors performed in duplicates, ***P ≤ 0.001. nd = under the limit of detection. Scale bars = 100 μm; nd = under the limit of detection. scale bars are the same for A–D and F–I. For all immunohistochemical analyses negative controls omitting the primary antibodies were performed resulting in the absence of the specific signals (data not shown).

We first tested endochondral ossification as a potential bone forming route for NC. Using a previously established protocol for the production of engineered hypertrophic cartilage tissues using BMSC 13, expanded NC were cultured as pellets in chondrogenic medium followed by hypertrophic induction. After chondrogenic induction, the resulting tissues displayed clear cartilaginous features, including positive Safranin-O staining for glycosaminoglycans (GAG) (Fig.1A) and large cells in lacunae embedded in abundant, mineral free matrix positive for COLL II (data not shown). Subsequent hypertrophic induction produced areas of enlarged cells (Fig.1B) with associated mineral deposition (Fig.1C) and positively stained for COLL X (Fig.1D). The expression of genes encoding for the above proteins matched the immunohistochemical findings and paralleled the expression profiles found in BMSC undergoing the same culture regime, including up-regulation of BSP and COLL-10 (Fig.1E). Analysis of the pathways involved in endochondral ossification on cells from the hypertrophy-induced constructs showed up-regulation of IHH, PTH1R and GLI1 to levels approaching those reached by BMSC and clearly distinct from AC (Fig.1E).

Implantation into subcutaneous pockets of nude mice of both chondrogenic- and hypertrophy-induced constructs (*n* = 3) yielded similar results at the analysed time-points. Hypertrophic explants underwent remodelling *in vivo*, resulting after 5 weeks in a marked decrease in Safranin-O staining intensity and of positivity for COLL X immunostaining (Fig.1F). After 12 weeks *in vivo*, NC-based constructs consistently established a GAG-rich cartilage tissue (Fig.1G) negative for COLL X (data not shown), in contrast to BMSC-based constructs which proceeded throughout endochondral ossification as previously described (Fig.1I) 13. Successful endochondral bone remodelling is known to hinge upon the vascularization of the implanted construct 21, whereas CD31 staining of explanted NC-based constructs demonstrated a total lack of vessel invasion from the host vasculature (Fig.1H). Additionally, the mRNA expression of chondromodulin, a potent anti-angiogenic factor specific to cartilage 22, was strongly up-regulated in NC during chondrogenic differentiation and hypertrophic induction. This was in contrast to BMSC, which maintained a statistically significant lower expression level throughout hypertrophy (Fig.1J). In summary, we demonstrated that NC can acquire a phenotype characteristic of hypertrophic cartilage under *in vitro* stimulation, but then revert back to stable chondrocytes upon *in vivo* implantation in a subcutaneous environment.

### Intramembranous ossification of NC in a subcutaneous environment is inefficient

**Figure 2:**
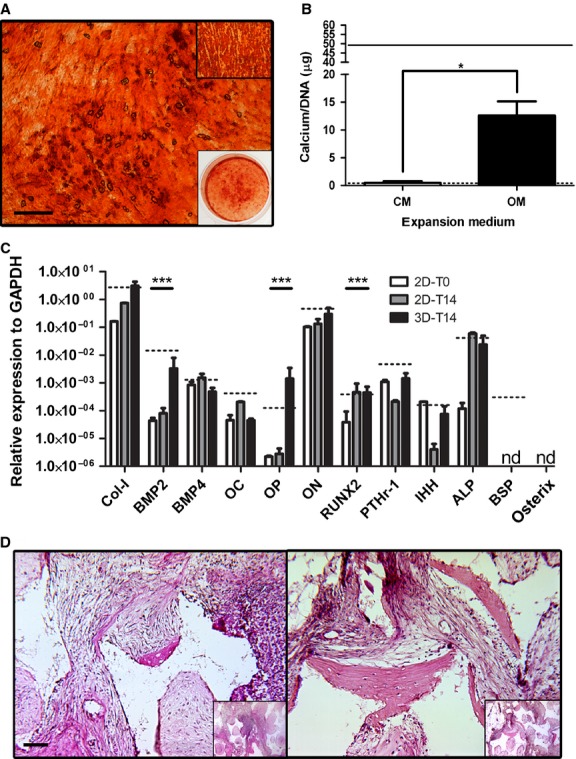
NC can osteoblastically differentiate, but do not efficiently proceed through intramembranous ossification in a subcutaneous environment. (A) Alizarin red staining of NC following osteogenic culture conditions (upper right box = BMSC under same conditions; lower right box = low magnification of whole well NC; scale bar = 1 mm). (B) Quantification of calcium matrix deposition of NC in complete medium (CM) and osteogenic medium (OM). Solid and dotted black lines represent calcium values for BMSC and AC, respectively, when cultured in OM. *P < 0.05; *n* = 3. (C) Gene expression levels of NC after expansion, after 2 weeks osteogenic induction in a monolayer (2D-T14) and after 2 weeks osteogenic induction in a 3D construct (3D-T14). Dotted line represents BMSC expression values at 3D-T14 time-point. nd = under the limit of detection. ***P < 0.001; represented significant differences noted only for selected markers of osteoblastic differentiation comparing 2D-T0 to 3D-T14; *n* = 3. (D) Haematoxylin and eosin staining of NC (left; one of only two bone ossicles detected) and BMSC (right) explanted 3D constructs after 8 weeks *in vivo*. Inserts are low magnification images of the entire constructs; scale bar = 100 μm.

We next analysed the capacity of NC to differentiate directly towards an osteoblastic phenotype *in vitro* and then undergo intramembranous ossification upon implantation *in vivo*. Expanded NC were exposed to osteogenic stimuli in either monolayer cultures (2D) or after embedding within ceramic-fibrin materials (3D). In 2D cultures, accumulation of mineral deposits in the extracellular matrix reached levels similar to those of BMSC (Fig.2A). Quantification of calcium per cell deposited onto the matrix was consistent with the intensity of alizarin red staining and indicated that osteogenically cultured NC could mineralize the ECM to an intermediate level between AC and BMSC (Fig.2B). Osteogenic stimulation in 2D culture resulted in the up-regulation of osteoblast-related genes, *e.g*. RUNX2 and ALP. Up-regulation of further osteoblastic genes, *e.g*. BMP2, OP and COLL-1, occurred in the 3D culture system, although BSP and osterix levels remained undetectable, indicating that *in vitro* osteogenically differentiated NC did not become fully functional osteoblastic cells. There was also an up-regulation of PTH1R and IHH in 3D constructs (8 and 10 times, respectively), but expression levels remained over 4 orders of magnitude lower than those seen during hypertrophic induction (Fig.2C), suggesting minimal hedgehog pathway activity. Implantation of 3D constructs (*n* = 3) after osteogenic differentiation into subcutaneous pockets of nude mice resulted in limited bone formation (only 2 scattered small bone regions) in only 1 of the 3 donors tested, while BMSC resulted in robust bone formation (reproducible presence of several frank bone areas in each construct; Fig.2D). We then decided to test whether this inefficient production of bone was a result of a limited fraction of cells present in the whole NC population that would be capable of osteogenic differentiation and bone matrix deposition.

### Clonal strains of NC have heterogeneous osteoblastic differentiation capacities

**Figure 3:**
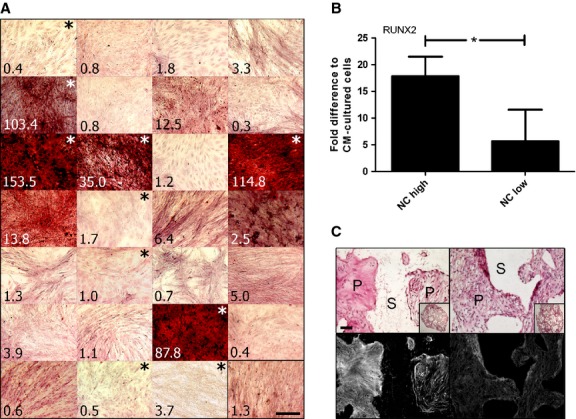
Clonal populations representing elevated intradonor heterogeneity and osteogenic capacity within a whole NC population. (A) Alizarin red staining of matrix obtained after 2 weeks osteogenic induction of all the individual clones from total population of NC. Numbers in bottom left of each image refer to total calcium (μg Ca/μg DNA) associated with a duplicate well. Lower right black-boxed image represents whole NC population. (B) qPCR of RUNX2 expression in NC clonal populations following osteogenic stimulation; *n* = 4. (C) Representative haematoxylin and eosin (upper row) and red filter florescence imaging of haematoxylin and eosin stained tissues to show relative collagen fibril densities (lower row) of explanted clones; Left column = NC high; right column = NC low. NC high = clones with intense alizarin red staining (indicated with white asterisk); NC low = clones with little to no alizarin red staining (indicated with black asterisk). S = decalcified scaffold (no cells present), P = pores of the scaffold (presence of cells and tissue development); scale bars = 100 μm.

A clonal study was performed to investigate the percentage of cells with osteogenic capacity present within the whole extracted NC population. The colony forming efficiency of NC, here defined as the percentage of plated single cells able to form a colony, was 36.5%. During the extensive expansion phase, including two passages, NC clones maintained an average proliferation rate of 0.90 ± 0.16 doublings/day, resulting in an average of 22.6 ± 1.1 total doublings. Cells isolated from the individual colonies were cultured in 2D in osteogenic medium. Mineralization capacity of the individual NC clonal populations was highly heterogeneous, as assessed by alizarin red staining and quantification of the amount of deposited calcium (Fig.3A). Expression analysis of the master gene for osteoblastic differentiation (RUNX2) 23–25 showed a positive correlation between its up-regulation and the mineralization capacity of the clonal strains (Fig.3B). Upon combination with fibrin-ceramic granules and implantation into subcutaneous pouches of nude mice, variances in matrix density were observed between implanted clones. In particular, clonal populations expressing higher amounts of RUNX2 *in vitro* resulted in higher tissue densities *in vivo* (Fig.3C). However, no individual clone was able to generate frank bone tissue.

### NC participate in bone formation in an orthotopic environment

**Figure 4:**
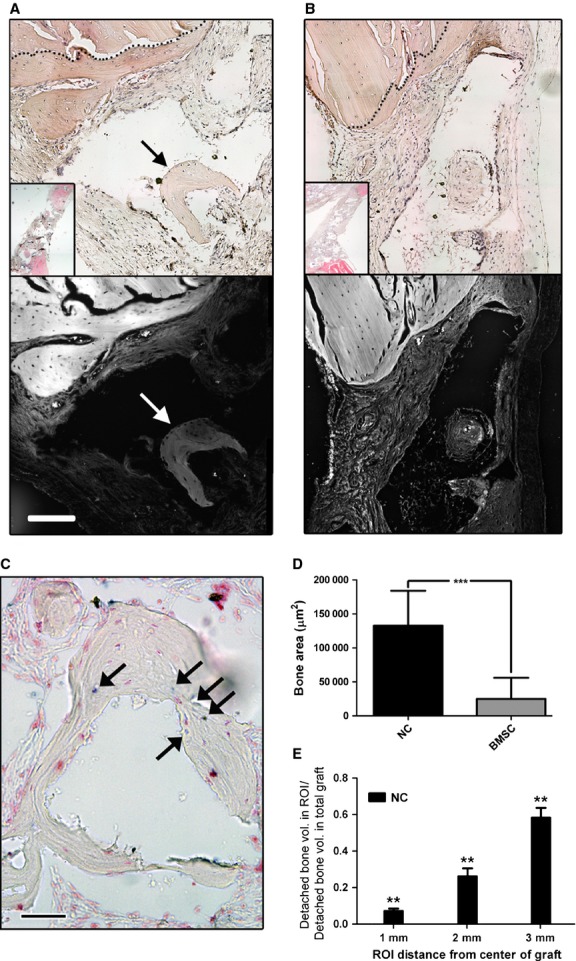
NC participate in bone formation in calvarian bone defects. Haematoxylin and eosin staining of explanted NC-based (A) and BMSC-based (B) constructs; Bottom images for both A and B are red filter florescence imaging of haematoxylin and eosin stained tissues to show relative collagen fibril densities; scale bar = 1 mm. Black and white arrows in A indicate detached bone ossicles. (C) *In situ* hybridization of human specific Alu sequences in an NC-based explanted construct; black arrows point to human nuclei; scale bar = 100 μm. (D) Histological section-based quantification of total detached bone volume; *n* = 3. (E) MicroCT-based quantification of detached bone formation (osteogenesis) 8 weeks after implantation into calvarial defects, demonstrating decreasing bone formation towards the centre of the construct. MicroCT was performed as described by Scotti *et al*. 13. Values are means ± SD of *n* = 9 per group (for each of the 3 implants per group, 3 regions were quantified). Significant differences from one experimental group to both other groups is indicated; **P < 0.01. ROI = region of interest.

We next hypothesized that an orthotopic environment could be necessary to support the NC towards more efficient bone formation. We thus implanted osteogenically induced NC and BMSC (*n* = 3) in 3D constructs into bilateral calvarial defects for 8 weeks. Upon explantation, two types of bone formation were observed: ingrowth bone from surgical margins, indicative of osteoconduction, and *detached bone*, here defined as ossicles without osseous link to the ingrowth bone, indicative of osteogenesis (Fig.4A and B). Human cells were found throughout the repair tissue, as assessed by Alu sequence staining, and also within the areas of *detached bone* (Fig.4C and Fig. S1). Quantification of *detached bone* volume areas in explanted constructs revealed an average of over 5 times more bone formation by NC than by BMSC (Fig.4D). Micro-CT analysis indicated that, while bone was found to be present even in the centre of the constructs, total *detached bone* volume decreased with the distance from the defect edges (Fig.4E). This finding suggests that the construct itself was not intrinsically osteoinductive, but the soluble signals from the native bone defect were responsible for the osteoblastic switch of the NC (Fig.4E). The result could be alternatively explained by an increased mortality of NC in the inner implant regions, as a result of lack of nutrients at the time of implantation. Interestingly, no BMSC were detected in the explants, whereas NC remained viable and were present throughout the *de novo*-formed tissue within the explants (Fig. S1).

## Discussion

This study provides a proof-of-principle that NC can directly convert into bone cells and actively participate in bone tissue formation. This was not observed in ectopic implantation models, even following hypertrophic or osteoblastic *in vitro* priming or clonal selection, but critically required a craniofacial, orthotopic *in vivo* environment. In that model, the efficiency of survival and bone formation by NC was significantly higher than that of reference osteogenic cells, namely BMSC.

Considering NC normally form a cartilage tissue *in situ*, we first wanted to determine if they could proceed through endochondral ossification. The phenotype acquired by NC after *in vitro* hypertrophic induction resembled that of BMSC, not only including COLL X deposition, but also the acquisition of a genetic signature known to be reflective of endochondral ossification 13. While BMSC could effectively form a functional bone organ *in vivo* after *in vitro* stimulation to a hypertrophic phenotype 26, under the same conditions NC did not develop into bone, but rather formed a stable cartilage tissue. The inability of NC to continue through the endochondral ossification route suggests that typical markers associated with endochondral bone formation (*e.g*., COLL X and IHH) 27 are not sufficient to establish the process. Considering that vascularization is critically required for endochondral bone formation *in vivo* 21, our finding could be related with the elevated production by NC of chondromodulin, a strong anti-angiogenic factor implicated in blocking hypertrophic cartilage vascularization and subsequent tissue turnover to bone 28.

Direct *in vitro* osteogenic stimulation of NC demonstrated that not only could NC produce a mineralized extracellular matrix just as effectively as BMSC, but that NC acquired a genetic profile similar to BMSC under the same conditions. In 3D culture, NC up-regulation of BMP2 and OP, combined with downregulation of the chondrogenic factor BMP4, is consistent with the osteoblastic differentiation profiles previously reported in neural crest progenitors 29. IHH was not up-regulated during osteogenic differentiation, consistent with the findings that its expression by neuroectodermally derived pre-osteoblasts inhibits further osteoblastic differentiation 30. In a subcutaneous implant environment, NC were capable to form frank bone tissue at a very low efficiency, suggesting that either the *in vitro* pre-osteogenic commitment was not stable enough to have a relevant *in vivo* effect or that only a small proportion of cells were competent to become fully functional osteoblasts. A clonal study designed to address the latter hypothesis demonstrated that none of the clonal strains implanted ectopically resulted in bone formation. However, these findings could have been intrinsically biased by the extensive population doublings underwent by the clonal populations (average of 22) as compared to the typical ones (average of 4.5) required for expansion of a whole NC population. Extensive proliferation has in fact been associated with the loss of *in vivo* osteogenic differentiation capacity in other mesenchymal precursor cell systems 31,32. Although the clonal experiments designed to explain the limited efficiency were not conclusive, the results of the multiclonal implantation proved that NC have the capacity to form frank bone tissue by direct osteoblastic differentiation, in contrast to the previously targeted route of endochondral ossification. Indeed, Calloni *et al*. postulated that bone cells from the craniofacial region arise from specific progenitors distinct from the osteochondrogenic, endochondral like ones found in long bones 25, thus further supporting a preferential capacity of NC to undergo intramembranous ossification.

To induce a more efficient direct osteoblastic transformation of NC, we next tested the cues present in an orthotopic environment. The cranial defect model used in this study reflects not only a bone environment that forms and heals through intramembranous ossification 33–35, but also a mixed embryological origin site providing homotopic (neuroectodermally- and mesodermally derived) signals for both cell types implanted (NC and BMSC) 36–38. Using this model we demonstrated, for the first time to the best of our knowledge, that a population with a previously considered stable chondrocytic phenotype could be converted into osteoblastic cells forming frank bone tissue *in vivo*. The behaviour of NC is different from that of AC, reported to form stable cartilage when implanted into calvarial defects 39, and could be reminiscent of embryonic neural crest-derived cells, which supported bone healing in a cranial defect upon differentiation into chondrocytes 40,41. Unexpectedly, under our experimental conditions, NC-based constructs outperformed BMSC-based ones in terms of osteogenesis and survival in the repair tissue. Indeed while large numbers of human NC could be detected in the repair tissue, no living BMSC could be observed. Obviously we cannot exclude that very few BMSC did survive, in which case their contribution to bone formation would likely be appreciated only after longer *in vivo* time. Mankani *et al*. in fact reported that bone formation by human BMSC in the calvarial defects of rats occurred at time-points superior to 8 week 42. Collectively, these results indicate that the implanted BMSC have a positive influence on vascularization and osteogenesis primarily by the release of trophic factors 43–45 as opposed to the survival after acquisition of an osteoblastic phenotype. Instead, the direct contribution of NC to bone formation, at least in the assessed time frame, demonstrates an intrinsically different mode of action, possibly related to the homotopic neuroectoderm nature of the cell origin and implantation site 46,47. Further studies will be necessary to investigate which environmental cues are responsible for the osteogenic induction of NC at the orthotopic and homotopic site, as well as to further characterize whether or not only specific subpopulations of NC can be effectively converted into osteoblasts. Indeed, it cannot be excluded that bone tissue was formed in our model by undifferentiated mesenchymal progenitors, either included within the nasal cartilage or – despite meticulous separation from the perichondral membrane – derived from a contaminant population from surrounding tissues.

A cell-based therapy has the potential to accelerate and improve the repair efficiency of critically sized craniofacial defects, but an adequate cell source has yet to be identified 48. Common cell-based strategies rely on BMSC or apical bone-derived osteoblasts, both of which display limited repair capacities 49 and associated drawbacks (low yield, donor site morbidity and high intradonor variability). NC could represent a homotopic cell source accessible under minimally invasive conditions and possibly used in autologous settings. Autologous NC have recently been clinically used for the reconstruction of the alar lobule 50 and are currently being tested for the treatment of articular cartilage defects at the University Hospital Basel (clinicaltrials.gov identifier: NCT01605201) 51. Their demonstrated ability to directly convert into a functional bone cell provides a proof-of-principle that the same cells may be further investigated in pre-clinical models for bone regeneration.

## Supporting Information

**Figure S1** *In situ* hybridization for human specific Alu sequences demonstrating the large presence of remaining (A) NC after 8 weeks calvarian implementation *versus* no detected presence of (B) BMSC under the same implant regime.
