# Supplementary figures and images for: Bone-forming capacity of adult human nasal chondrocytes
Source: J Cell Mol Med. 2015 Feb 16;19(6):1390–9. doi: 10.1111/jcmm.12526 (PMC4459852; doi:10.1111/jcmm.12526)

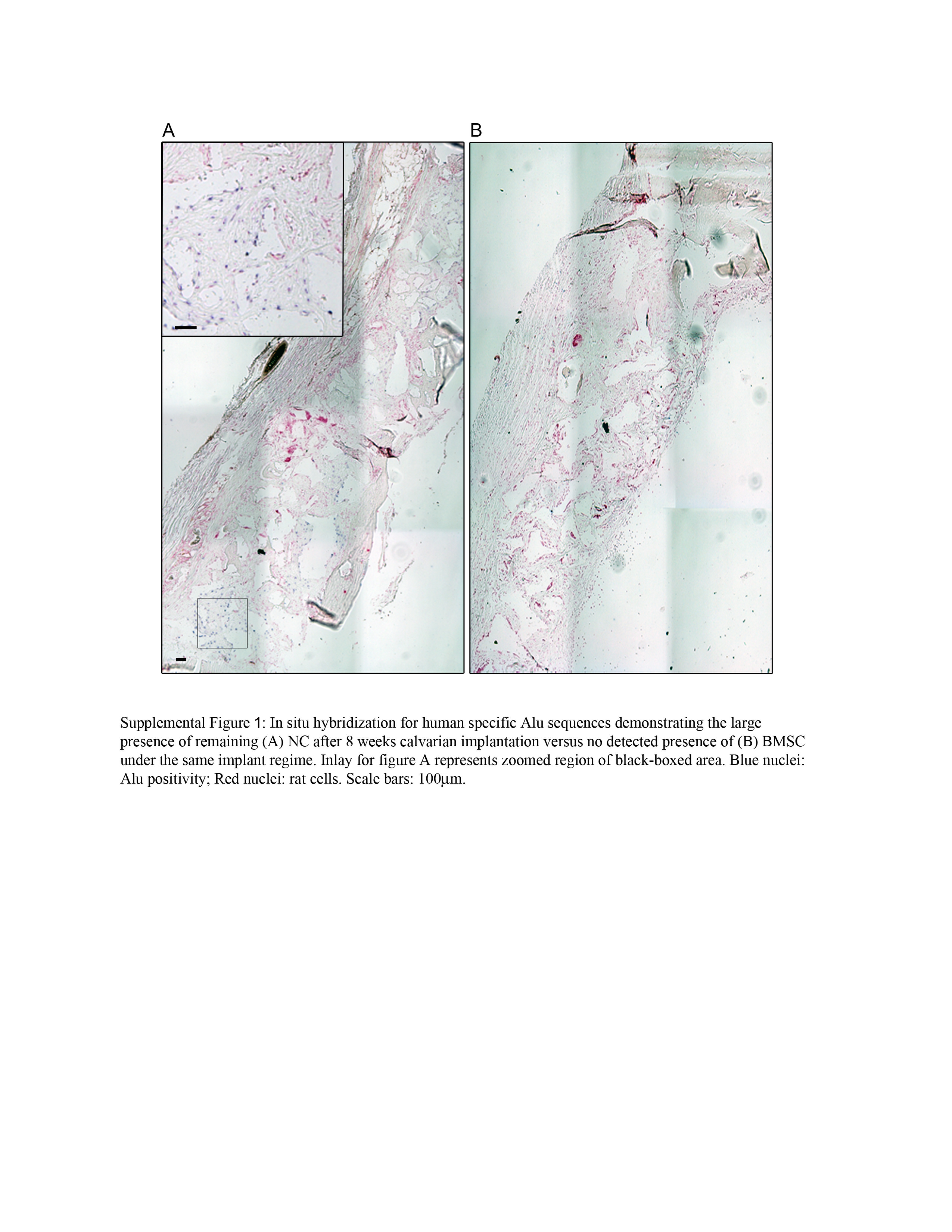

Supplement: Supplementary file 1 [file jcmm0019-1390-sd1.tif]
